# Congenital Cutis Laxa: A Case Report and Literature Review

**DOI:** 10.3389/fsurg.2022.814897

**Published:** 2022-03-16

**Authors:** Yang Kun, Shi Mengdong, Fu Cong, Huo Ran

**Affiliations:** ^1^Department of Plastic Surgery, Shandong Provincial Hospital, Cheeloo College of Medicine, Shandong University, Jinan, China; ^2^Department of Plastic Surgery, Shandong Provincial Hospital Affiliated to Shandong First Medical University, Jinan, China

**Keywords:** Congenital Cutis Laxa, plastic surgery, recurrence, systematic treatment, sequential treatment

## Abstract

Cutis Laxa is a rare connective tissue disease featuring inelastic and saggy skin. It is thought that plastic surgery might be the most effective treatment, while the previous pieces of literature on the surgical treatment for Cutis Laxa complained of the recurrence. We report a patient of Congenital Cutis Laxa who has received systematic and sequential treatment based on plastic surgery. The patient is content with the effect of treatment, and there are no signs of recurrence after 5 months. By referring to relevant pieces of literature, we evaluate the clinical manifestations and diagnosis of the disease. A multi-step, systematic, and sequential treatment is recommended for the treatment of Congenital Cutis Laxa.

## Introduction

Cutis Laxa (CL) is a group of rare connective tissue diseases characterized by lack of skin elasticity, sagging, with or without variable systemic involvement. CL manifests in acquired and congenital forms. Acquired Cutis Laxa usually occurs in early adulthood (1, 2), while congenital Cutis Laxa (CCL) is often symptomatic at birth or during infancy and shows a high degree of inheritance heterogeneity. As a rare disease, there is a small number of pieces of research on the treatment of CCL, and many people believe that plastic surgery might be the most effective treatment (3). We report a case of a patient of Congenital Cutis Laxa, who complained of systemic skin relaxation from 3 days after birth. The patient showed a typical “hound-dog” appearance feature, and his gene detection has proved autosomal dominant. The patient has performed facial rhytidectomy in June 2021, and is satisfied with the effect of the operation. By referring to relevant pieces of literature, retrospectively analyzing the diagnosis and treatment process, we evaluated the clinical manifestations, diagnosis, choice of treatment methods and post-treatment of the disease, and summarized the application value of plastic surgery treatment in this disease.

## Case Report

**Figure 1:**
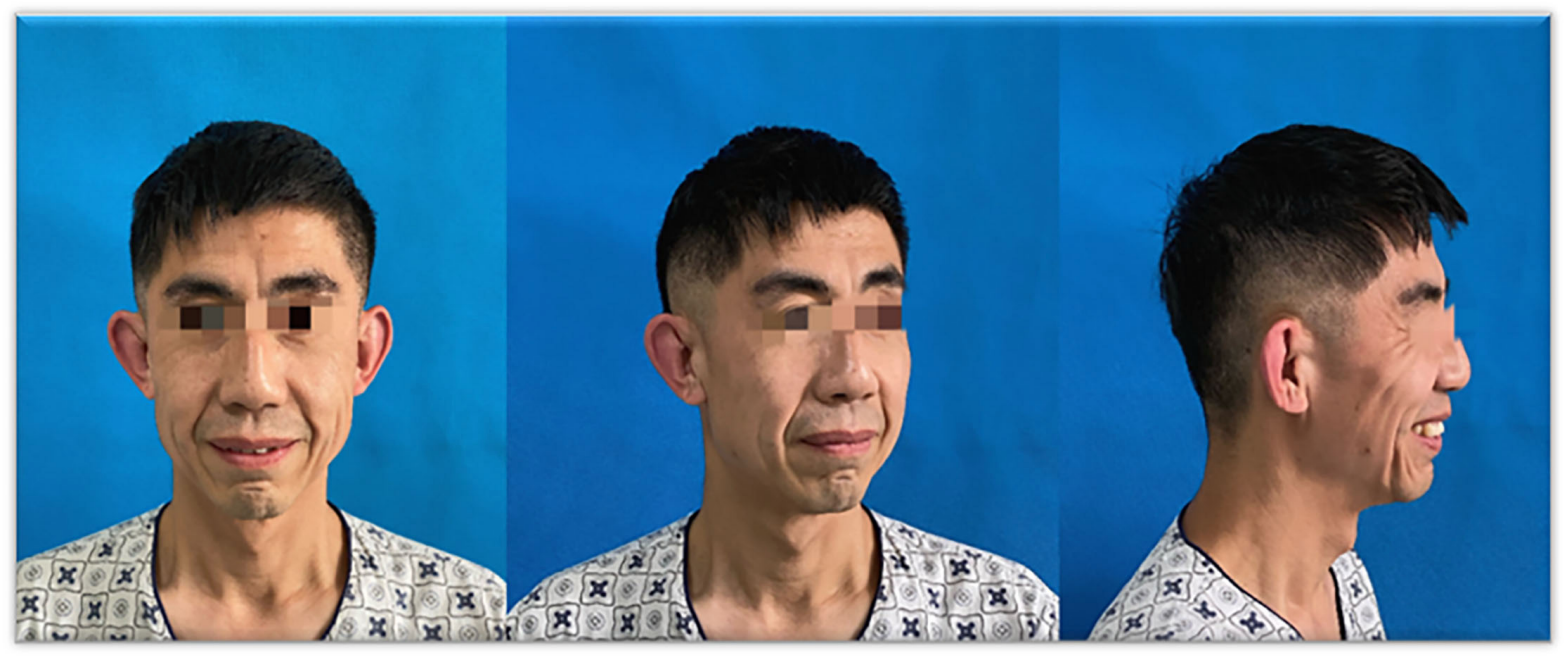
Pre-operative frontal and oblique views.

A 26-year-old man was admitted to hospital for being conscious of facial skin inelastic and saggy over the 26 years. The patient complained of systemic skin relaxation from 3 days after birth, especially the middle and lower parts of the face, as shown in Figure 1. With growth and development, the general sagging condition of the patient is becoming better than before.

### Previous History

The patient is the second child and full-term spontaneous delivery. His mother denied the history of toxic radiation exposure during pregnancy and childbirth. The patient's parents and sister were in good health, and the parents denied close marriage. The patient had been operated on the inguinal hernia at the age of 6 years old, and post-operative scar was not obvious. The patient denied having family history of hereditary diseases and infectious diseases.

### Physical Examination

The skin of the whole body is inelastic and saggy, especially the middle and lower parts of the face, with external canthus ptosis, nasal tip collapse, long and soft earlobes and the lower part folding, philtrum shallow, deep nasolabial sulcus, and ptosis of labial angle, showing a typical “hound-dog” appearance feature.

### Laboratory and Ultrasound Examination

Fibrinogen, 1.88 g/L [normal, 2.00 ~ 4.00 g/L]. No abnormalities were found in liver, gallbladder, pancreas, spleen, and kidney. The heart structure was roughly normal.

### Pathological Examination

There was neutrophil infiltration around small vessels in the dermis of skin tissue. Elastic fiber staining showed that elastic fibers were reduced and broken. Elastic fiber (+).

### Gastroenteroscopy

Ectopic gastric mucosa of esophagus. Reflux esophagitis. Esophageal Iscan staining, non-atrophic gastritis with erosion (HP infection).

### Gene Detection

One mutation of ELN gene was found, and the associated disease was autosomal dominant Cutis Laxa type 1 (OMIM: 123700). The related clinical features are skin relaxation and hernia.

### Diagnosis

Congenital Cutis Laxa.

### Treatment

**Figure 2:**
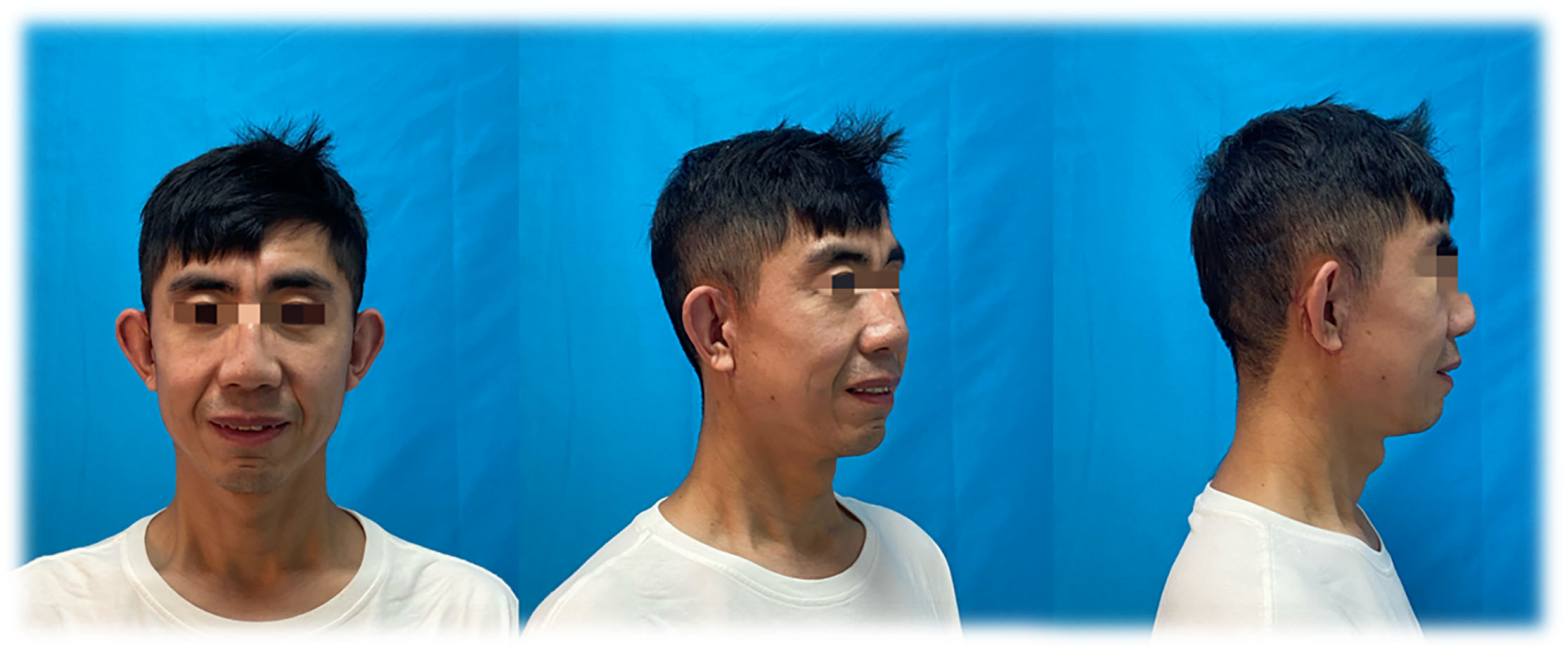
Post-operative frontal and oblique views for 5 months after the surgery.

Complete relevant examinations and exclude surgical contraindications. The surgical was performed under general anesthesia. Design surgical incision from the front of the tragus to behind the auricle. After finishing routine disinfection and setting sterile towels, use 2% lidocaine, saline, and epinephrine to swell and infiltration anesthetize the operative region. After several minutes, incise skin to superficial fascia along the marked surgical incision. Along the superficial musculo-aponeurotic system (SMAS), the dissection is done towards the midline, up to the zygomatic arch and cheeks. After completing hemostasis, remove 2-cm skin within the incision line, and repair a skin edge. Use 5-0 PDSII to do the local intermittent suture in the SMAS layer, and use 5-0 PDSII intermittent suture subcutaneously, 6-0 single-silk nylon suture closed skin for the surgical incision area. Disinfect the incision with saline and alcohol in sequence, then cover with sterile dressings. The excision tissue was set for pathology. The patient has used scar cream and worn pressure masks for at least 1 month, and then received 755-nm non-ablative fractionated laser therapy and dietary guidance. The patient is content with the effect of treatment, and there are no signs of recurrence after 5 months, as shown in Figure 2.

## Discussion

As a rare connective tissue disease, Cutis Laxa is characterized as loose skins with decreased resilience and elasticity, and with or without systemic involvement. CL manifests in acquired and congenital forms. Acquired Cutis Laxa (1, 2) shows different degrees of pathological changes in the structure and quantity of elastic fibers due to the application of certain pathogenic factors to the skin, and ultimately leads to the pendulous and lax skin localized or generalized. Congenital Cutis Laxa (CCL) shows a high degree of inheritance heterogeneity and, often, is symptomatic at birth or during infancy. As the patients of CCL grow older, the saggy skin is usually lessened than before.

**Figure 3:**
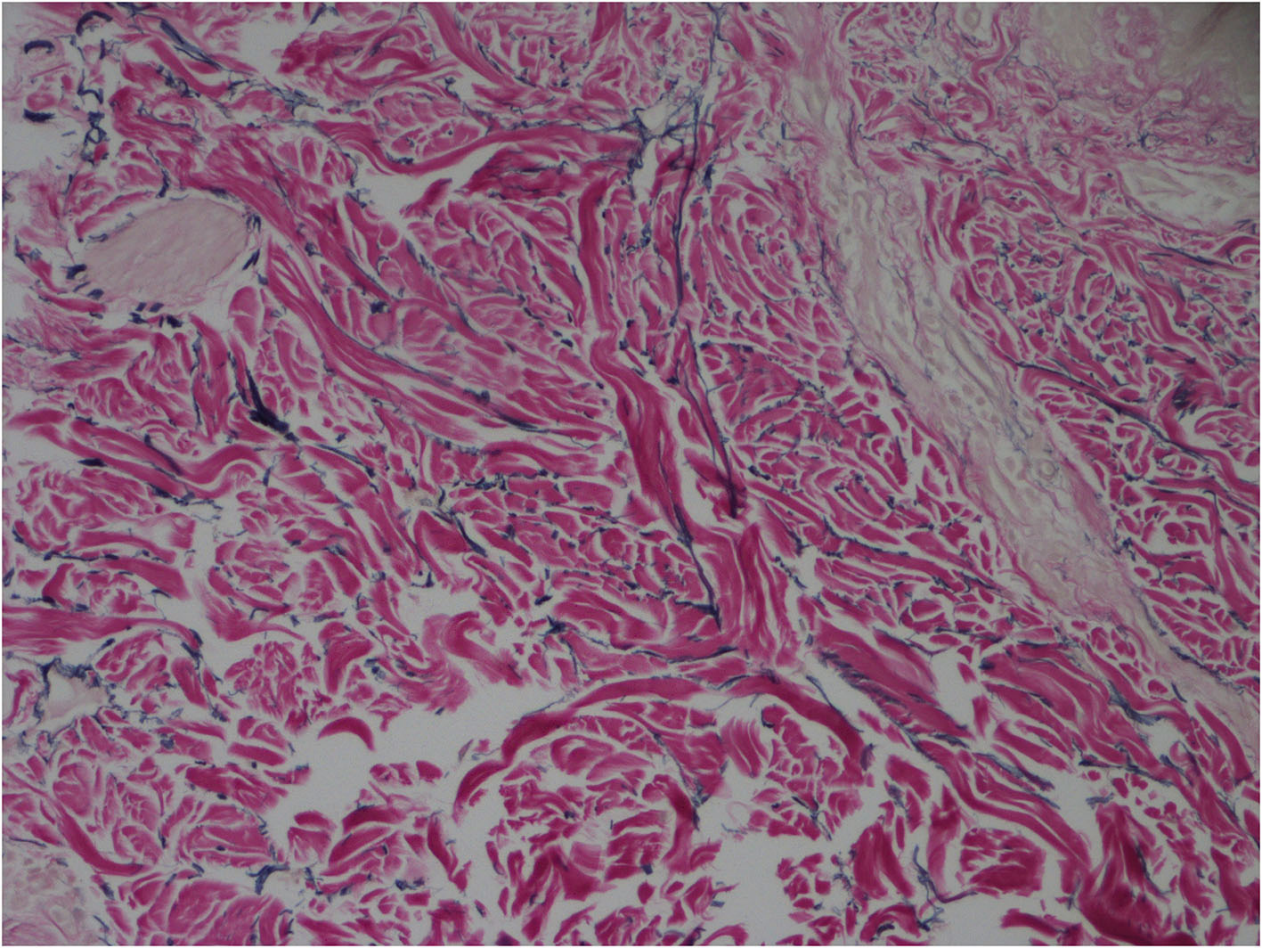
Absence or significant reduction of elastic fibers in the dermis layer and residual fragments of elastic fibers (elastic fiber staining × 400).

The major pathological manifestations of CCL are absence or significant reduction of elastic fibers in the dermis layer and residual fragments of elastic fibers after elastin staining (4), as shown in Figure 3. There, also, might be perivascular inflammatory infiltration dominated by lymphocytes, microthromboses (5) or phagocytosis of elastic fibers by macrophages (6), while the pathogenesis of CCL is still unclear (7). The potential mechanisms might be the upregulation or decreased inhibition of elastolytic enzyme activity, ischemia, phagocytosis of elastic fibers, and autoimmune and inflammatory processes, which lead to the increasing breakdown of elastic fibers. According to the different genetic modes and gene mutation sites, CCL is subdivided into 5 subtypes internationally (8, 9), which are X-linked genotype, autosomal-dominant genotype, autosomal-recessive genotype I (ARCL1), autosomal-recessive genotype IIA (ARCL2A), and autosomal-recessive genotype IIB (ARCL2B). The patient we reported had undergone genetic detection and proved autosomal-dominant genotype of CCL. The gene detection used EDTA anticoagulated peripheral blood of the patient to finish the all-exome detection in a proband mode. The detection was divided into three main steps, which was using high-throughput sequencing technology to finish the mutation screen, and then using bioinformatics and clinical information analysis technology to analyze genetic data, and using Sanger sequencing technology to verify the suspected pathogenic mutation. At last, this detection found one mutation of ELN gene, which chromosomal location is Chr7:74063683 and nucleic acid change is c.1985delG, as shown in Table 1, and the verification of genetic variation is an exchange between C-G base pairs. According to ACMG guidelines (2015), this mutation of ELN is pathogenic, and highly matches with the clinical feature of CCL and inguinal hernia (10). Combining the medical history with clinical manifestations can make a preliminary diagnosis, while the definite diagnosis should be based on pathological examination and genetic detection. Genetic detection can clarify the genetic mode of CCL, predict the disease development, and provide fertility guidance for patients.

**Table 1:** The genovariation of patient.

**Gene**	**Chromosomal location**	**Nucleic acid changes**	**Amino acid changes (Variant No.)**	**RS No**.	**MAF**	**Pathogenic levels of ACMG**	**Index case (male)**	**Related diseases (OMIM No.), hereditary mode**
ELN	Chr7:74063683	c.1985delG	p.G662Afs*25 (NM_000501.4)	None	Not included	Pathogenic	Heterozygote (68/159)	Autosomal dominant Cutis Laxa type 1(OMIM: 123700), AD; Supravalvular aortic stenosis (OMIM:185500), AD

It is highly important to grasp the surgical indications of CCL and should be fully checked before treatment to exclude contraindications and to differentially diagnose from other hyperelasticity diseases, which have poor skin healing, such as Ehlers-Danlos syndrome or Hutchinson-Gilford progeria syndrome (11). Ehlers-Danlos syndrome (EDS) is a group of genetic connective tissue disorders, which are characterized by skin hyperextensibility, tissue fragility joint, and hypermobility (12). Patients with ED syndrome have poor skin healing, so they are not suitable for plastic surgery. The skin in patients with EDS is hyperextensible and quickly recovers from distension, while, in CCL, the skin is loose and recovers slowly from distention; this difference can clinically differentiate EDS from CCL (13). What is more, in the skin pathology section of patients in ED syndrome, collagen fibers are loose and disordered, while, in CCL, the collagen fibers are basically normal, which can be used as a standard for discriminating diagnosis. Hutchinson-Gilford progeria syndrome, also known as progeria, is a rare and fatal disease, which is characterized by accelerated aging from early childhood, and, eventually, people die in the second decade because of myocardial infarction or stroke (14). Those patients suffer from not only the aging skin appearance but also progressive developing diseases, including atherosclerosis, lipoatrophy, skeletal dysplasia, alopecia, and other fetal diseases (15). The pathogenic mutation of LMNA gene in chromosome 1q causes this kind of degenerative disorder (16). One of the functions of LMNA gene is to encode prelamin A, and then prelamin A converts into lamin A, and lamin A plays a crucial role in stabilizing the nuclear membrane as a nuclear lamina structural protein constitution. The patients of Hutchinson-Gilford progeria syndrome have characteristic facial appearance of micrognathia, prominent eyes, and circumoral cyanosis, alopecia, and prominent scalp veins. Moreover, those patients present sclerotic skin changes and a reduced joint range of motion, which can distinguish Hutchinson-Gilford progeria syndrome from CCL in clinical appearance.

There is a small number of pieces of research on the treatment of CCL, and the existing treatment methods include plastic surgery, laser (17), intralesional administration (18), radiofrequency devices, and so on. However, the most effective treatment method is considered to be the plastic surgery. Surgical treatment can produce the desired result immediately and maintain the effect significantly longer than the others. The other treatments are unclear efficacy, although there have been some case reports proving improvement of the general sagging condition after receiving those treatments. By searching PubMed, Cochrane Library, Embase, Web of Science, Wanfang, HowNet, Weipu, and other databases, using “Congenital Cutis Laxa” and (or) “surgical treatment” and other keywords to search related pieces of literature, we included 5 case reports (3, 9, 19–21) on the surgical treatment of CCL. Those 5 case reports presented 7 patients of CCL who had received different kinds of rhytidectomy. Among those reports, 5 patients suffered from other diseases such as inguinal hernia and so on, and 5 patients complained of recurrence after surgery for several months, and 2 of them received more than two-time surgeries. The facts that most patients are usually concomitant with other systemic diseases and have a high probability of recurrence after surgery remind us of choosing plastic surgery as the only treatment with caution, even though plastic surgery is currently considered to be the most effective treatment (2).

We developed a multi-step diagnosis and treatment plan for our patient to carry out a systematic and sequential treatment. Before operation, the patient must complete relevant examinations, including abdominal ultrasound and cardiac ultrasound, to exclude surgical contraindications. And then we communicated with the patient about the risks during the operation and the high possibility of recurrence. What is more, psychological counseling may be of great help for the patient. After the operation, we followed up the patient regularly and instructed him to use scar cream and wear pressure masks to reduce scar hyperplasia. Moreover, laser therapy and dietary guidance in an outpatient clinic were recommended. Cho (17) proved that treatment with a 10,600-nm carbon dioxide fractional laser system might be effective in improving skin texture. Wang (22) recommendes the combination treatment with a 595-nm pulsed-dye laser and a 1,550-nm non-ablative fractionated laser. The potential mechanism in laser therapy is related to the regeneration of elastin fibers. Combined, the application of laser therapy and surgery may be more effective in long-term result. By dietary guidance, a moderate increase in weight can fill the facial skin and reduce the condition of sagging from the appearance. The patient was satisfied with the treatment effect, and the incision heals nicely, which fits in with a cosmetic standard, and there are no signs of recurrence after 5 months.

In summary, we recommend systematic and sequential treatment based on plastic surgery for the treatment of CCL. Although multiple surgeries may be required, the social and psychological benefits need to be considered, and early surgical intervention is recommended. Due to the small number of cases and the lack of long-term follow-up, the accuracy of systematic and sequential approach selection and patient prognostic evaluation need to be further studied and demonstrated.

## Data Availability Statement

The original contributions presented in the study are included in the article/Supplementary Material, further inquiries can be directed to the corresponding authors.

## Ethics Statement

Written informed consent was obtained from the individual(s) for the publication of any potentially identifiable images or data included in this article.

## Author Contributions

YK performed the majority of the writing. FC reviewed the literature and contributed to manuscript drafting. HR and FC were responsible for the revision of the manuscript for important intellectual content. SM analyzed and interpreted the pathology findings. YK and SM made the literature review. All authors issued final approval for the version to be submitted.

## Conflict of Interest

The authors declare that the research was conducted in the absence of any commercial or financial relationships that could be construed as a potential conflict of interest.

## Publisher's Note

All claims expressed in this article are solely those of the authors and do not necessarily represent those of their affiliated organizations, or those of the publisher, the editors and the reviewers. Any product that may be evaluated in this article, or claim that may be made by its manufacturer, is not guaranteed or endorsed by the publisher.
