# Supplementary material for: Congenital Cutis Laxa: A Case Report and Literature Review
Source: Front Surg. 2022 Mar 16;9:814897. doi: 10.3389/fsurg.2022.814897 (PMC8966123; doi:10.3389/fsurg.2022.814897)
Supplement: Supplementary file 1 [file Data_Sheet_1.docx]

**Table 2** Summary of Case Report Data

| Case report | Gender, age | family history | previous history | Treatment methods | Follow-up ending |
| --- | --- | --- | --- | --- | --- |
| Li Wuyan et al. | F，17 | deny |  | Rhytidectomy for temporal buccal cervical | Recurrence after 20 months |
|  | F，10 | yes | inguinal hernia | Rhytidectomy for temporal buccal cervical | Recurrence after 24 months |
|  | M，13 | deny |  | Rhytidectomy for temporal buccal cervical | Recurrence after 6 months |
| Thomas W O, et al. | F，10 | Yes | descending aortic dilatation | rhytidectomy and nasolabial excision | satisfaction |
| Nia D, et al. | F，48 | deny | Emphysema;  severe hypertension; bilateral renal artery fibromuscular dysplasia | rhinoplasty and excision of excess skin from the upper arm at the age of 16；rhytidectomies with excision of excess skin at the age of 20； rhytidectomy at the age of 37；rhytidectomy with superficial musculoaponeurotic system plication at the age of 42 | Repeated recurrence |
| Nahas F X, et al. | F，23 | deny | inguinal hernia | rhytidectomy and blepharoplasty for 2times | recurrence within 6months at the first surgery |
| Tas A, et al. | F，12 | deny | cross eyelids,  watery eyes, alternating esotropia | Bilateral lateral canthal tendon repositioning and bilateral medial rectus recession procedures |  |
